# Beyond Oncolytics: *E1B55K*-Deleted Adenovirus as a Vaccine Delivery Vector

**DOI:** 10.1371/journal.pone.0158505

**Published:** 2016-07-08

**Authors:** Michael A. Thomas, Tinashe Nyanhete, Iskra Tuero, David Venzon, Marjorie Robert-Guroff

**Affiliations:** 1 Section on Immune Biology of Retroviral Infection, Vaccine Branch, National Cancer Institute, National Institutes of Health, Bethesda, Maryland, United States of America; 2 Biostatistics and Data Management Section, National Cancer Institute, National Institutes of Health, Bethesda, Maryland, United States of America; University Hospital of Navarra, SPAIN

## Abstract

Type 5 human adenoviruses (Ad5) deleted of genes encoding the early region 1B 55-kDa (E1B55K) protein including Onyx-015 (*dl*1520) and H101 are best known for their oncolytic potential. As a vaccine vector the *E1B55K* deletion may allow for the insertion of a transgene nearly 1,000 base pairs larger than now possible. This has the potential of extending the application for which the vectors are clinically known. However, the immune priming ability of *E1B55K*-deleted vectors is unknown, undermining our ability to gauge their usefulness in vaccine applications. For this reason, we created an *E1B55K-deleted* Ad5 vector expressing full-length single chain HIV_BaL_gp120 attached to a flexible linker and the first two domains of rhesus CD4 (rhFLSC) in exchange for the *E3* region. In cell-based experiments the *E1B55K*-deleted vector promoted higher levels of innate immune signals including chemokines, cytokines, and the NKG2D ligands MIC A/B compared to an *E1B55K* wild-type vector expressing the same immunogen. Based on these results we evaluated the immune priming ability of the *E1B55K-deleted* vector in mice. The *E1B55K*-deleted vector promoted similar levels of Ad5-, HIVgp120, and rhFLSC-specific cellular and humoral immune responses as the *E1B55K* wild-type vector. In pre-clinical HIV-vaccine studies the wild-type vector has been employed as part of a very effective prime-boost strategy. This study demonstrates that *E1B55K-*deleted adenoviruses may serve as effective vaccine delivery vectors.

## Introduction

To facilitate the goal of an effective preventative HIV vaccine, we are developing an approach involving priming with replicating adenovirus (Ad) recombinants and boosting with envelope protein. This approach has elicited potent transgene-specific humoral and cellular immune responses [1] capable of affording protection against HIV, SIV, and simian/human immunodeficiency virus (SHIV) challenges in rhesus macaque and chimpanzee models [2–7]. Based on these results, a prime-boost approach involving replicating Ad sub-type 4 (Ad4*ΔE3*) is being developed for both HIV and influenza vaccines [8–10]. Replicating Ad5 with deletions in addition to the E3 region have other clinical uses. In Ad5 where the *E1B55K* gene is deleted a defect allows the vector to replicate in and selectively kill cancer cells. Thus *E1B55K*-deleted vectors such as Onyx-015 (*dl*1520) have made it to phase 3 clinical trials in the US as cancer therapeutics. A similar vector (H101) has been released to treat cancer in China [11, 12]. *E1B55K*-deleted vectors may be suited for vaccine delivery purposes as well. Such vectors may permit the insertion of larger transgenes and/or immune modulators not currently possible with the *E1B55K* wild-type vector, however their immune priming ability remains unknown. To address this, we created a replication-competent *E1B55K-/E3*-deleted Ad5 host-range mutant (Ad5hr)-recombinant encoding full-length single chain HIV_BaL_gp120 attached to a flexible linker and the first two domains of rhesus CD4 (rhFLSC). This rhFLSC HIV immunogen was previously described [13] and used in the *E1B55K* wild-type vector for immunization of mice and nonhuman primates [14, 15]. These experiments showed the wild-type vector to be very effective at promoting HIVgp120 and rhFLSC-specific immune responses. Therefore, if an *E1B55K*-deleted vector were able to promote similar specific immune responses as the wild-type vector, then it might stand as a better vaccine delivery vector.

## Results

### The *E1B55K*-deleted vector promotes higher levels of innate immune signals than the *E1B55K* wild-type

**Fig 1:**
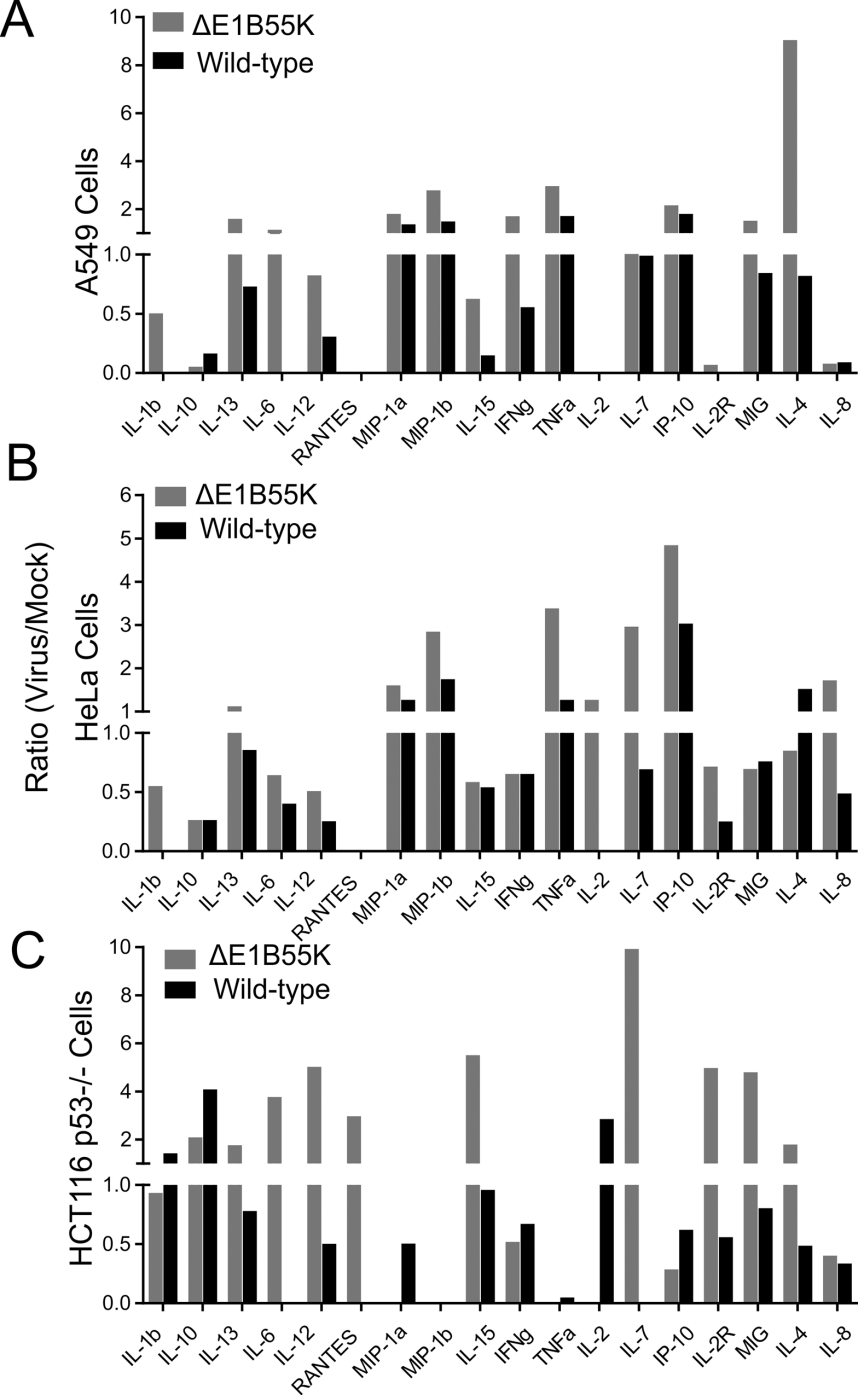
*E1B55K*-deleted Ad5 vector promotes higher levels of chemokines/cytokines than the E1B55K wild-type vector. Multiplex ELISA assay using **(A)** A549 **(B)** HeLa or (**C**) p53- HCT116 cells following mock- or Ad-infection. An MOI 50 for each virus was used to infect the A549, and 200 for HeLa and p53-HCT116 for 24–48 hours. The ratio of values obtained after subtraction of the background from the virus infections relative to those obtained for the mock-infected cells are shown.

Ad5 E1B55K is reported to control expression of immune response genes [16]. Therefore, a vector bearing an *E1B55K* deletion may display innate immune signals that differ from an *E1B55K* wild type vector. In Fig 1, as in most of our results, we anchor our experiments to HeLa cells as most of what is known about Ad is in the context of this cell line. The multiplex ELISA experiments show differences in levels of individual chemokines/cytokines produced by Ad5-infected A549 (Fig 1A), HeLa (Fig 1B), and the HCT116 cells lacking p53 (p53-/-; Fig 1C). Overall, the levels of chemokines/cytokines were consistently higher in all three cell lines infected with the *E1B55K*-deleted virus compared to those infected with the *E1B55K* wild-type virus.

### Ad5 infections stimulate surface expression of NKG2D ligands

**Fig 2:**
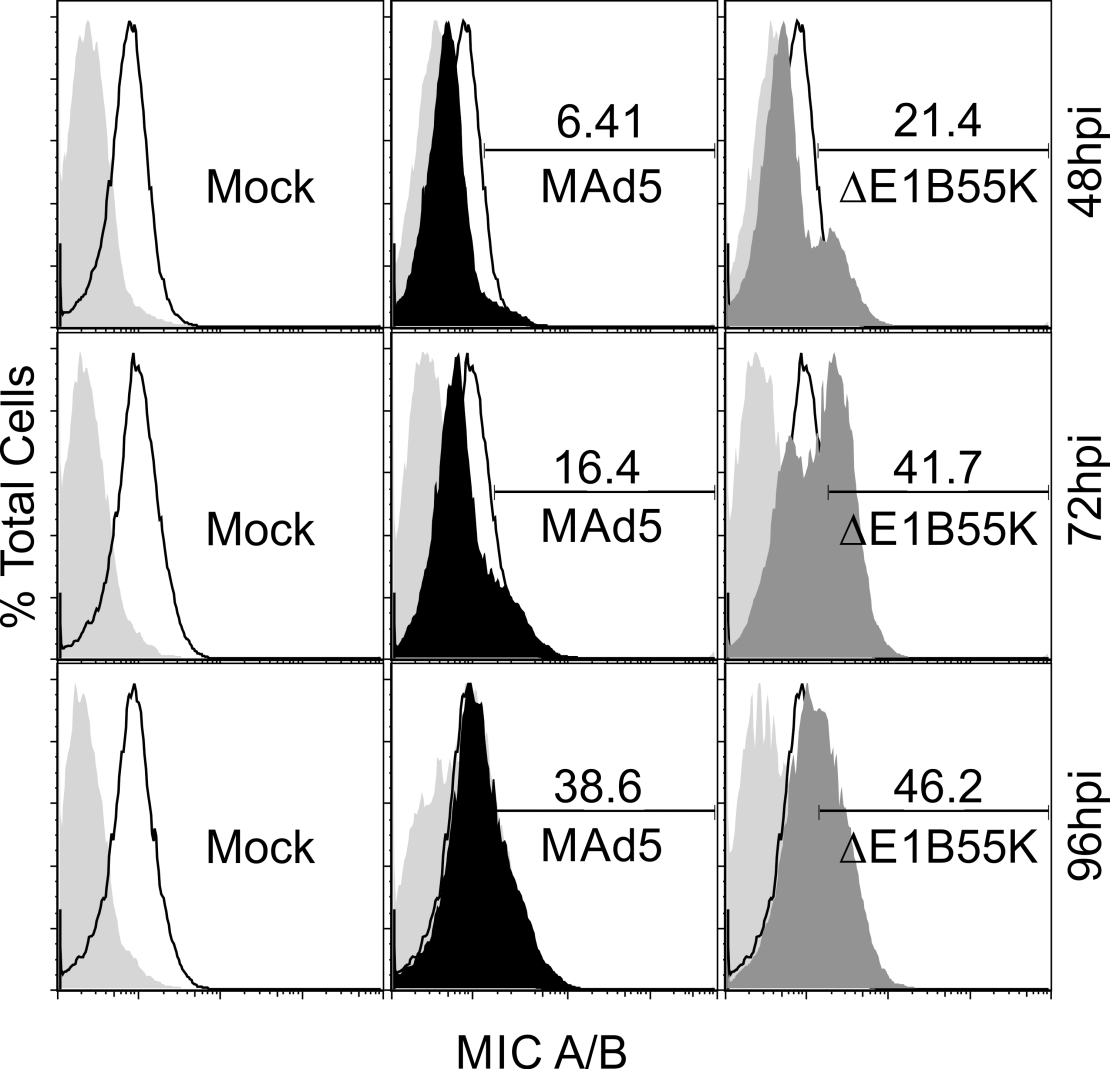
Ad5 increases the expression of NKG2D ligands in infected cells. HeLa cells were mock-infected (open histogram) or infected with equal concentrations of MAd5rhFLSC (MAd5, black histogram) or *ΔE1B55K*rhFLSC (ΔE1B55K, dark gray histogram) for 48, 72 or 96 hours. Cells were surface stained with an isotype control (light gray histogram) or anti-human MIC A/B antibodies. Gates based on the positive mock-infection were copied to the other histograms to obtain percent MIC A/B positive cells. Results are representative of 4 to 5 independent experiments.

Ad5 E1A has been reported to enhance the expression of NKG2D ligands in human and mouse cells [17]. Yet an activity of the E319K protein (not in our vectors) is dedicated to preventing its surface expression [18]. This can only imply that the expression of NKG2D ligands occurs as an unintended outcome of the action of E1A. NKG2D is an activating receptor found on the surface of innate system cells including natural killer (NK) cells, cytotoxic CD8^+^ T cells, some CD4^+^ T cells and γδ T cells [19]. To determine the effects of the *E1B55K* deletion on NKG2D ligands we infected HeLa cells that are known to express MIC A/B with MAd5rhFLSC or *ΔE1B55K*rhFLSC. At early times post-infection Ad5 seems to repress surface expression of MIC A/B. By 48 hours post infection (hpi) surface expression levels of MIC A/B increased in a fraction of *ΔE1B55K*rhFLSC-infected cells to above that observed for both mock and MAd5rhFLSC-infected cells (Fig 2 row 1). Expression levels of the ligands continued to increase at 72 and 96 hpi (Fig 2 rows 2 and 3). From these experiments it appears that in addition to E319K, E1B55K may function, in part, to stave off the enhanced expression of NKG2D ligands in Ad-infected cells. Thus, in *ΔE1B55K*rhFLSC-infected cells, loss of *E1B55K* may lead to a variety of signals that alert the immune system to a virus infection.

### The *E1B55K*-deleted vector promotes lower levels of late viral proteins, progeny virions and HIV transgene than an *E1B55K* wild-type vector

**Fig 3:**
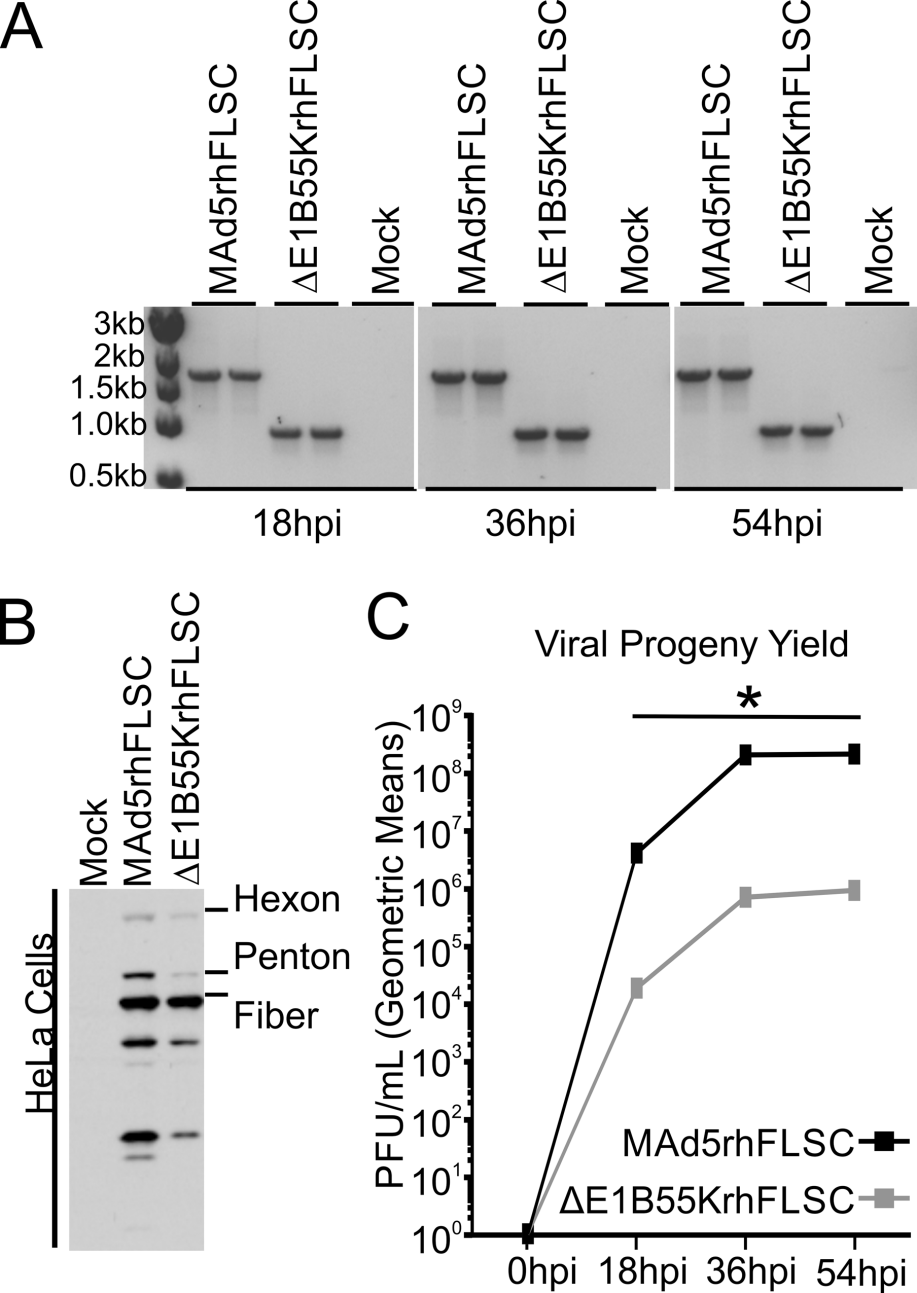
DNA, Ad5 protein and virus yield of the *E1B55K*-deleted vector *ΔE1B55K*rhFLSC. **(A)** DNA isolated from infected HeLa cells 18, 36, or 54 hpi yielded a 1759 base pair PCR fragment in MAd5rhFLSC-infected cells that was reduced to 827 base pairs in *ΔE1B55K*rhFLSC infected cells. **(B)** Western blot analysis using anti-Ad5 antibody shows that MAd5rhFLSC-infected HeLa cells produce higher levels of late viral proteins 24 hpi than those infected by the *ΔE1B55K*rhFLSC virus. The figure is representative of 4 experiments. **(C)** Media from part (**A**) were diluted and used in plaque assays. The geometric mean values of two independent infections performed in duplicate for each time point were compared using the stratified Wilcoxon rank sum test. * = p value <0.02.

We used PCR to confirm the *E1B55K* deletion. Because the primers spanned the *E1B* region, an 827 base pair (bp) band was observed in lanes containing DNA from the *ΔE1B55K*rhFLSC vector and a 1759 bp band in lanes containing DNA from the MAd5rhFLSC vector (Fig 3A). The E1B55K product is required for efficient late viral mRNA cytoplasmic accumulation [20, 21]. For that reason cells infected with vectors devoid of *E1B55K* produce lower levels of late viral proteins [20–22] as shown here (Fig 3B). Levels of progeny virions were also significantly lower (over 2.0 logs, p = 0.019) in the *ΔE1B55K*rhFLSC-infected HeLa cells compared to those infected with the *E1B55K* wild-type virus, MAd5rhFLSC (Fig 3), consistent with results obtained with *E1B55K*-deleted vectors shown elsewhere [21–23].

**Fig 4:**
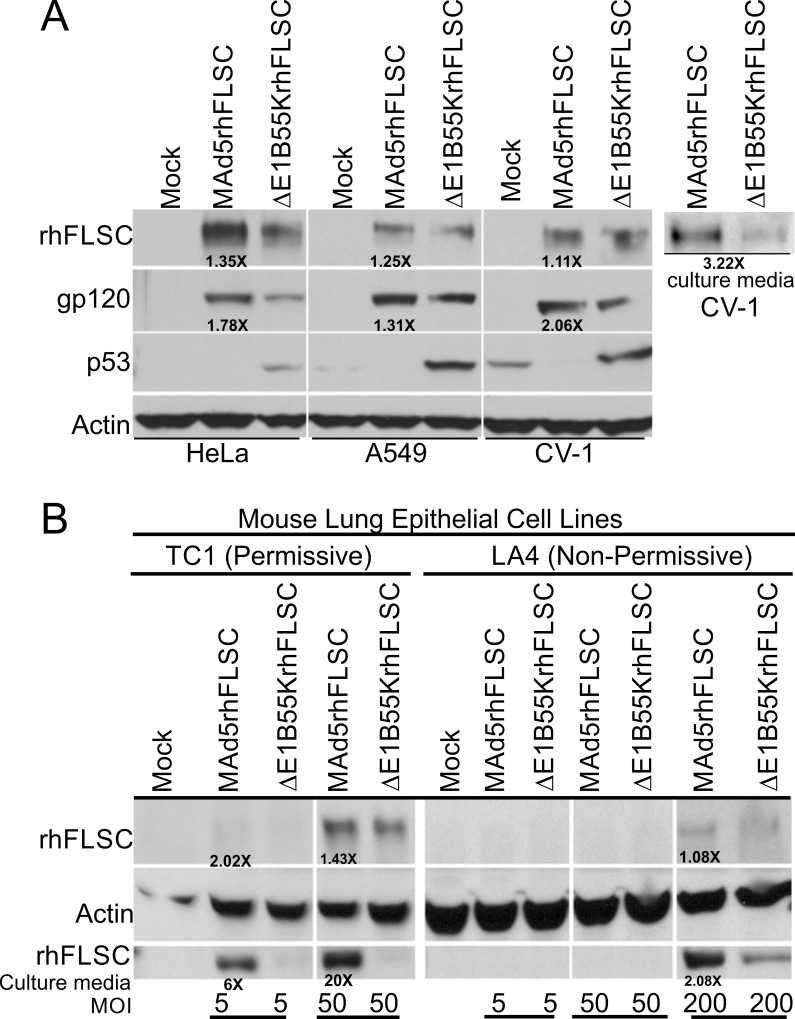
Transgene expression profiles of the *E1B55K*-deleted vector *ΔE1B55K*rhFLSC. (**A**) HeLa, A549, CV-1, (**B**) TC1 and LA4 cells were mock-infected or infected with MAd5rhFLSC or *ΔE1B55K*rhFLSC at various MOI ranging from 5–200 for either 24 or 48 hours. The cells were lysed by boiling in 1X protein sample buffer and equivalent amounts were analyzed by western blot using anti-CD4 (for rhFLSC), anti-gp120, anti-p53 or anti-actin as indicated. Cell growth media collected from (**A**) CV-1, (**B**) TC1 and LA4 cells were boiled in 1X protein sample buffer and equivalent amounts were analyzed by western blot using anti-CD4 (for rhFLSC). The figure shows a representative result of 2–4 experiments with actin-normalized fold increases.

We further assessed the contribution of the E1B55K deletion on expression levels of the HIV rhFLSC immunogen. Differences in levels of rhFLSC and gp120 were very noticeable in lysate from *ΔE1B55K*rhFLSC-infected HeLa and TC1 cells but less so in lysate from infected A549, CV-1 and LA4 cells (Fig 4). More apparent were differences in protein levels secreted into the media as shown here for the infected CV-1 cells and both mouse cell lines (Fig 4). It is not surprising that the levels of HIV rhFLSC/gp120 mirror those of Ad5 late proteins since expression of this immunogen is most likely governed like the Ad5 late proteins[14]. From these results, *E1B55K*-deleted Ad5-vectors promote lower levels of the HIV transgene than the *E1B55K*-wild type vector.

### The *E1B55K*-deleted vaccine vector induces cytokine producing HIVgp120 and rhFLSC-specific memory T-cells

**Fig 5:**
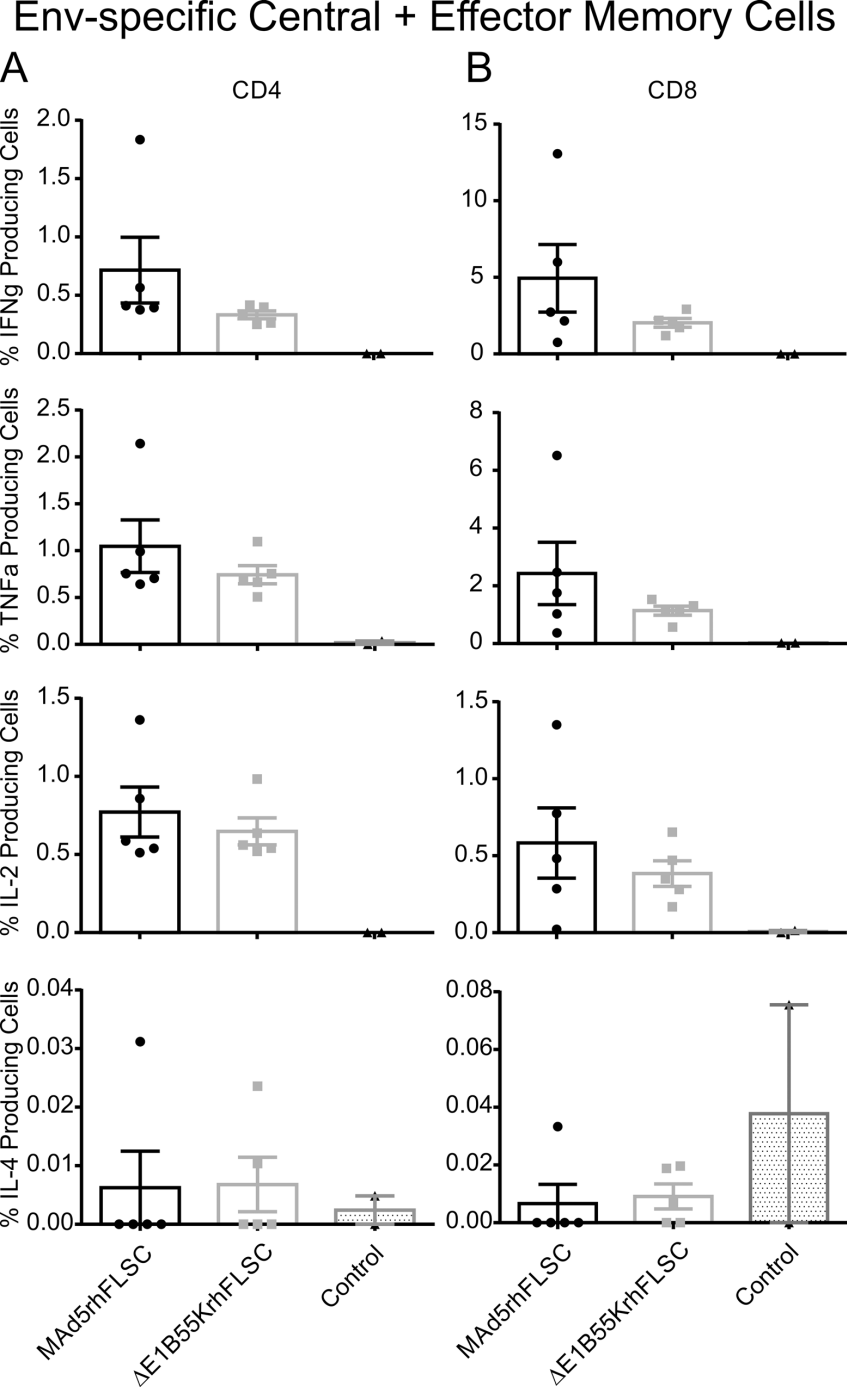
Mice immunized with an *E1B55K*-deleted vaccine vector produce similar levels of cytokine producing cells as those immunized with an *E1B55K*-containing vector. **(A-B)** Intracellular cytokine staining of splenocytes for Env-specific CD8 and CD4 central and effector memory T cells secreting IFNγ, IL-2, TNFα, and IL-4. **(A)** No differences were observed between the means of the two immunized groups for IFNγ, IL-2, or TNFα cytokine producing CD4 central and effector memory T cells. **(B)** No differences were observed between the means of the two immunized groups for IFNγ, IL-2, or TNFα cytokine producing CD8 central and effector memory T cells. The values for the control mice were zero for CD4 and CD8 IFNγ, IL-2, and TNFα producing central and effector memory T cells. **(A-B)** For IL-4 no differences were seen in the means of all three groups. Mean values are plotted with error bars indicating SEM. Differences were measured by Mann-Whitney-Wilcoxon test.

The increased levels of innate system signals seen in *ΔE1B55K*rhFLSC-infected cells suggested that this vector might be more immunogenic than Ad5 vectors wild-type for the *E1B55K* gene. In spite of the differences in rhFLSC expression levels we next assessed the potential contributions of the *E1B55K* deletion on Ad5 and HIV-transgene immunogenicity in immunized Balb/C mice as described previously [14]. In these experiments we used flow cytometry to interrogate splenocytes and thereafter compared frequencies of intracellular cytokine positive cells producing IFNγ, IL-2, TNFα and IL-4 in response to stimulation with HIV_Bal_gp120 peptides. Among CD44^high^ CD4^+^ cells, the proportions of cells expressing IFN-γ, TNF-α, and IL-2 were similar for the immunized groups, both of which exhibited significantly higher levels than the controls. (Fig 5A). One of the animals in the MAd5rhFLSC immunized group consistently had a greater percentage of cytokine-positive cells for reasons that are not understood. Similar results were observed for CD44^high^ CD8^+^ cells (Fig 5B). This was not the case for IL-4, where levels were negligible for all the groups (Fig 5A & 5B bottom row).

Attempts at measuring Ad5 fiber-specific T cell responses yielded only background values (not shown). In our previous study we observed only very low levels of Ad5-specific T cells in mice immunized with the MAd5rhFLSC vector [14]. Thus these results are consistent with our prior report.

### The *E1B55K*-deleted vaccine vector induced binding antibodies against Ad-, HIVgp120 and the rhFLCS-immunogen

**Fig 6:**
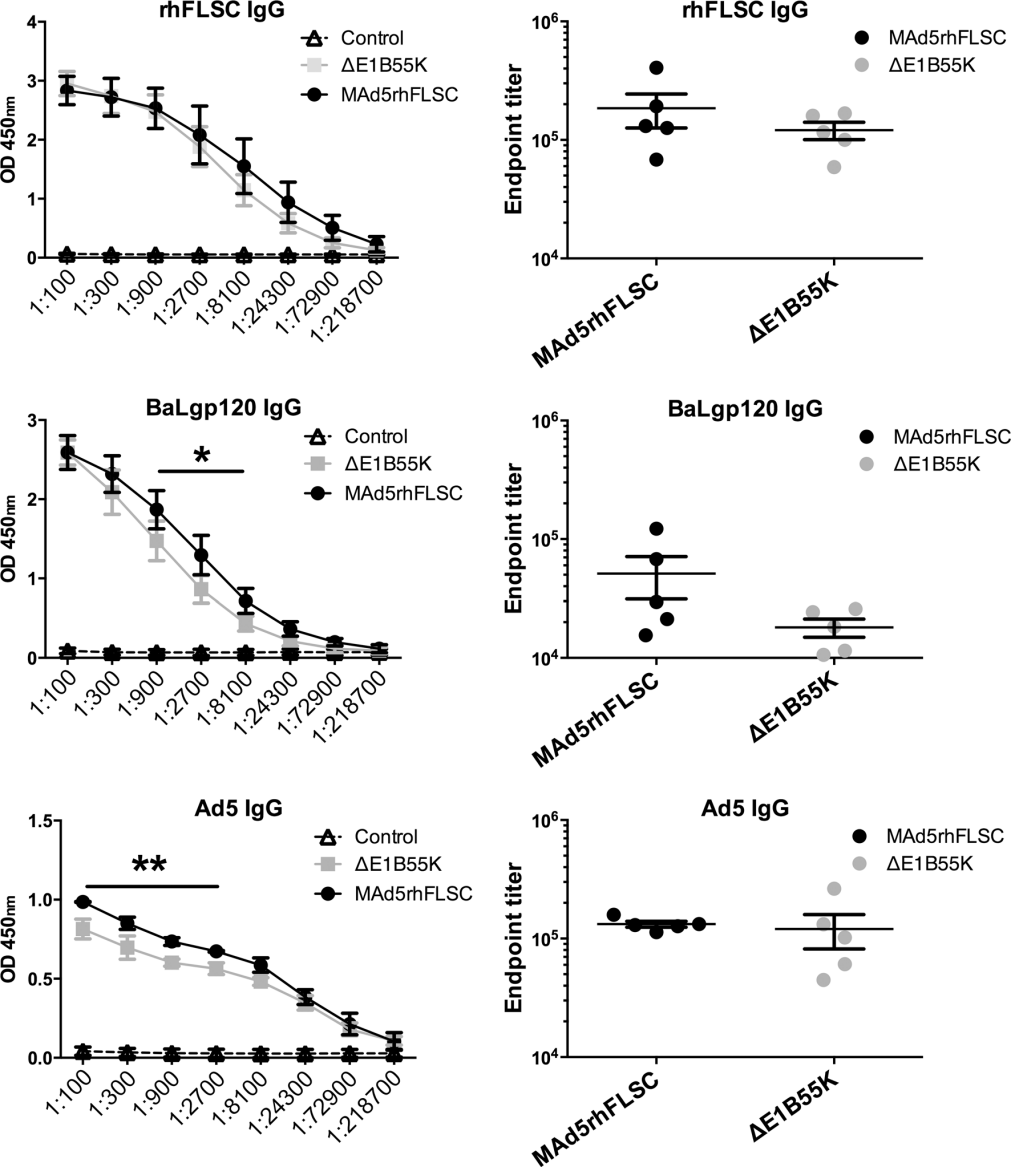
Mice immunized with an *E1B55K*-deleted vaccine vector develop similar IgG antibody titers to the transgene and Ad5 vector as those immunized with an *E1B55K*-containing vector. Sera collected from immunized mice were diluted and used in ELISA assays to determine binding to rhFLSC protein, HIV_BaL_gp120 protein or Ad5 particles. OD450 and endpoint titers for each mouse were plotted. No differences in IgG endpoint titers were observed between MAd5rhFLSC and the *ΔE1B55K*rhFLSC viruses for rhFLSC protein, HIV_BaL_gp120 protein or Ad5 particles. Higher binding induced by MAd5rhFLSC immunization against HIV_BaL_gp120 at intermediate serum concentrations (*, p<0.01) and against Ad5 at higher serum concentrations (**, p<0.001) was observed. Each experiment was performed in duplicate and repeated up to 4 times. Mean values plus SEM are plotted.

To determine the effects of *E1B55K* deletion on the levels of IgG binding antibodies induced against the vector and the HIV immunogen, sera from immunized mice were evaluated by ELISA. Both the *ΔE1B55K*rhFLSC and MAd5rhFLSC vectors induced high-titered binding antibodies against rhFLSC and the HIV_BaL_gp120 subunit (Fig 6 rows 1 & 2). The mean rhFLSC and gp120 endpoint titers of sera from both groups of immunized mice were significantly higher than the controls which were essentially zero. Higher binding (p < 0.01) to HIV_BaL_gp120 at intermediate serum concentrations was seen in the MAd5rhFLSC-immunized mice (Fig 6, row 2, left-hand panel). IgG binding antibodies against Ad5 elicited in the immunized mice were assessed by ELISA using plates coated with viral particles. No significant difference was observed between the endpoint titers of the two immunization groups (Fig 6, last row, right-hand panel) even though binding differed (p < 0.001) at higher serum concentrations (1:100 to 1:2700 serum dilutions; Fig 6, last row, left-hand panel). Overall, mice inoculated with MAd5rhFLSC and the *ΔE1B55K*rhFLSC vector elicited similar levels of antibodies.

### Indistinguishable levels of HIV- and Ad5-specific Ig isotypes

**Fig 7:**
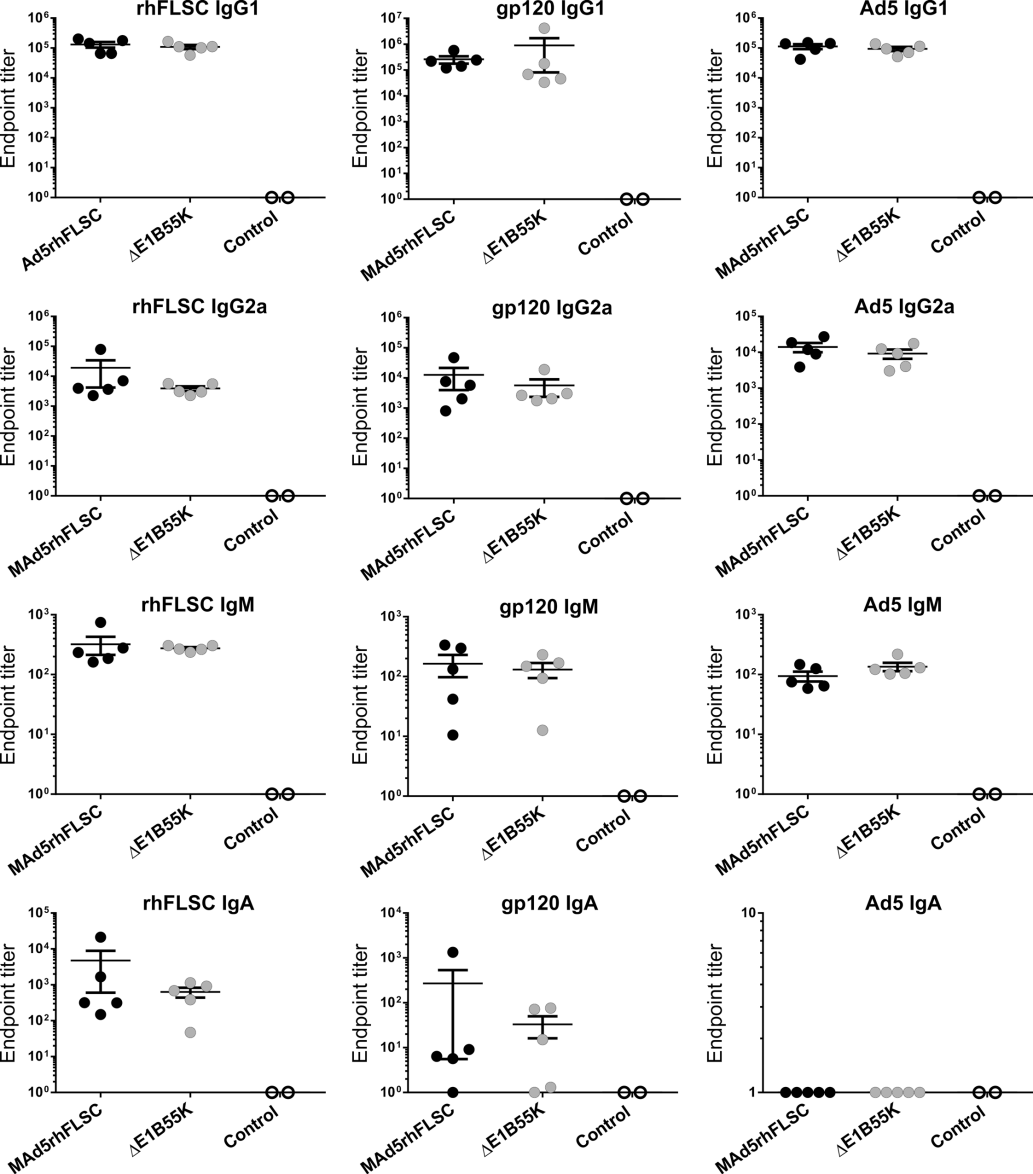
Ig-subtypes induced in mice immunized with *ΔE1B55K*rhFLSC. Sera collected from immunized mice were diluted and used in ELISA assays to examine IgG1 and IgG2a subtypes and Ig-isotypes produced against rhFLSC protein, HIV_BaL_gp120 protein or Ad5 particles. Mean endpoint titers plus SEM for each mouse are shown. No differences in endpoint titers were observed between MAd5rhFLSC and the *ΔE1B55K*rhFLSC viruses for rhFLSC protein, HIV_BaL_gp120 protein or Ad5 particles by ANOVA. Each experiment was performed in duplicate and repeated 1 to 4 times.

The specific IgG subtype induced against a pathogen may influence immunological function and determines whether it persists or is quickly cleared from the system [24]. Therefore, we investigated whether immunization with the *ΔE1B55K*rhFLSC vector may have altered levels of specific IgG subtypes elicited against rhFLSC, HIV_BaL_gp120, or Ad5 particles. In these experiments IgG1 predominated against both the HIV transgene and the Ad5 vector (Fig 7). This is in contrast to a previous report suggesting that IgG2a predominates against viral infectious agents [24]. Appreciable levels of IgG2a were also produced against the HIV transgene and the Ad5 vector but titers were approximately 10-fold less than those of IgG1 (Fig 7 row 2). Other Ig(s), IgM and IgA, were also detectable but at lower levels. It is interesting to note that no serum IgA was elicited against Ad5 antigens (Fig 7). It is possible that the IgA may have translocated to mucosal sites not evaluated in this study.

## Discussion

Our interest in the *E1B55K* deletion stems from a desire to generate additional carrying capacity in our replicating Ad5 vaccine vector. When added to the already deleted *E3* region and considering that Ad5 can stably package up to 105% of its genome [25], an *E1B55K/E3*-deleted vector should carry transgenes nearly 5.5kb in size. This would allow us to insert larger genes and/or immune modulators currently not possible with the *E1B55K* wild-type vector. An added benefit of *E1B55K*-deleted Ad5 is that they have been shown in numerous clinical trials to be safe for use in the human population [11, 12]. In fact, *E1B55K*-deleted vectors such as *dl*1520/Onyx-015 and H101 are recognized for their oncolytic potential [11, 12]. H101 is also presently used to treat cancer [11, 12]. Here we demonstrate yet another use for these viruses—that of vaccine delivery vectors.

It is possible that levels of rhFLSC promoted by the two vectors contributed to the outcome we observed in the immunized mice. An Ad5 immunogen engages the immune system in three ways: 1) by binding of the inoculum virus to B-cells, macrophages and or dendritic cells; 2) by viral proteins produced and secreted into the surrounding milieu; 3) by the uptake of infected dead or dying cells by surrounding cells. All of these lead to processing and antigen presentation. The transgene by contrast, only engages the immune system by the latter two mechanisms. Consequently, there might be a limit beyond which immunogen expression levels make no immunological difference. In that case the different levels of rhFLSC promoted by the two vectors (Fig 4) would not be expected to influence the immune responses we observed in the immunized mice. Alternatively, differences in virus-host cell interactions produced by the *E1B55K* deletion might have stimulated Ad- and HIV-specific innate and adaptive immune responses that compensated for the increased levels of immunogen promoted by the wild-type virus. Indeed, differences in peptide and protein immunogen concentrations as little as 3- to 5-fold have led to detectable changes in immune responses [26, 27]. For that reason, we expected the *E1B55K* wild-type vector to produce measurably higher immune responses in the immunized mice. That it did not supports the latter possibility. This possibility is strengthened by the fact that in cluster analysis genes encoding immune responses were enriched in *E1B*-mutant viruses [16] supporting a role for the E1B55K protein in inhibiting Ad-induced inflammation [28]. While the link between E1B55K and transgene immunogenicity remains to be further explored, it is worth pointing out that here the mouse model used removed the replicative property of the vectors. Thus any differences observed were solely a consequence of the viral gene products. In a species permissive for Ad5 replication, it is possible that the greater replicability of the *E1B55K* wild-type virus might offset the greater immunogenicity of *E1B55K-*deleted vectors. This remains to be explored.

We noted differences in binding of IgG to HIV_BaL_gp120 and Ad5 in the immunized mice (Fig 6, rows 2 and 3, left panels). This suggested possible differences in one or all of the specific Ig-subtypes and/or isotypes induced against the transgene or Ad5 vector. However, our results (Fig 7) showed no differences in titer of the 4 subtypes/isotypes evaluated. We were not able to assess IgG2b or IgG3 due to insufficient sera. Therefore, it remains possible that the quality of the antibodies produced by the two vectors differed.

Finally, even while our demonstration of the transgene-specific immune priming ability of *E1B55K*-deleted Ad5 vectors was shown using an HIV immunogen, there is no reason why the transgene could not be replaced by a cancer or another disease-specific immunogen. Henceforth we anticipate that *E1B55K*-deleted Ad5 will be increasingly recognized as a vaccine delivery vector.

## Materials and Methods

### Ethics Statement

This study was carried out in strict accordance with the recommendations in the Guide for the Care and Use of Laboratory Animals of the National Institutes of Health. The protocol (Protocol # VB-005) was approved by the NCI-Bethesda Animal Care and Use Committee (ACUC). All efforts were made to minimize suffering.

### Cell culture

All cell lines were obtained from the American Type Culture Collection. Cervical carcinoma-derived HeLa cells, human embryonic kidney-derived 293 cells, human lung adenocarcinoma epithelial derived A549 cells, and African green monkey kidney-derived CV-1 cells were maintained in Dulbecco’s modified Eagle’s medium (Life Technologies) supplemented with 10% fetal bovine serum (Life Technologies). Mouse lung epithelial cells TC1 and LA4 were maintained in RPMI (Life Technologies) supplemented with 10% newborn calf serum (HyClone Laboratories). Cells were maintained at 37°C in a humidified atmosphere with 5% CO_2_.

### Viruses

The MAd5rhFLSC virus (wild-type with respect to *E1B55K*) is described elsewhere [14]. To create *ΔE1B55K*rhFLSC, BamHI digested, gel isolated pBRAd5hr*ΔE3* (TPL-rhFLSC-pA) shuttle plasmids [14] were used as the right-hand part of the virus. The larger fragment of DNA isolated from EcoRI digested *dl*1520 [23] served as the left-hand part. The left- and right-hand fragments were co-transfected with lipofectamine 2000 (Invitrogen) into QBI 293 cells, incubated at 37°C and monitored for the presence of cytopathic effects (CPE). Viral DNA was isolated using a QIAamp DNA Blood Mini Kit (QIAGEN) and the resulting *ΔE1B55K*rhFLSC recombinant candidates were screened by PCR using the following forward and reverse primer pairs: TTTTCTGCTGTGCGTAACTT; ATCTTCATCGCTAGAGCCAA. This yielded a 1759 base pair fragment in the wild-type but an 827 base pair fragment in viruses lacking *E1B55K* (Fig 3A). Expression of Ad5 late proteins (Fig 3B) and the transgene rhFLSC (including the gp120 component) (Fig 4) was evaluated by Western blot. The recombinant viruses were further purified by three rounds of plaque purification. Aliquots of each pure viral stock were amplified on 293 cells and purified twice by cesium gradient centrifugation. The concentrations of the viral stocks were determined by optical density (OD) and plaque forming units (PFU) by plaque assays (SAIC, Fredrick, MD). The particle/PFU ratio for the *E1B55K*-deleted virus (6.0x10^10^ particles/5.0x10^10^ PFU) was 1.2 and for MAd5rhFLSC 1.3.

### Virus yield

Viral progeny yields (Fig 3C) were determined by plaque assay as described previously [22]

### Gel electrophoresis and western blot

Gel electrophoresis and western blot were performed as previously described [14] with the exception of those performed using cell growth media where cells were infected at a range of MOIs from 5 to 200. After 24 or 48 hours 50–100 uL growth media was collected and diluted in protein sample buffer (1X SDS Gel Loading Dye, 10% BME). Equal amounts of samples were run on 4–20% SDS-polyacrylamide gels (Life Technologies) and transferred to nitrocellulose membranes using the iBlot Western Blot System (Life Technologies). Blots were blocked in PBS with 0.02% Tween 20 and 5% milk for 2 hours and thereafter exposed to a 10% milk buffer containing one of the following primary antibodies at 4° overnight or for 2 hours at room temperature: anti-actin (Sigma-Aldrich); anti-p53 (BD Biosciences); anti-HIV-1 gp120 (Meridian Life Sciences); anti-hCD4 (R&D Systems); and anti-Ad type 5 (Abnova). Subsequently, the blots were washed and exposed to an HRP conjugated secondary antibody, either anti-mouse IgG, anti-human IgG, anti-rabbit IgG, or anti-goat IgG (KPL) as dictated by the primary isotype. Chemiluminescent detection was performed using SuperSignal West Pico Chemiluminescent Substrate (Thermo Fisher Scientific) or LumiGLO Chemiluminescent Substrate System (KPL).

### Animals and vaccination

Six- to eight-week-old female BALB/c mice were housed and maintained in a pathogen-free environment according to the standards of the American Association for Accreditation of Laboratory Animal Care at the NIH (Bethesda, MD). The animal protocol was reviewed and approved by the Animal Care and Use Committee prior to implementation. Mice (NCI Frederick, 5 per group) were inoculated intraperitoneally at weeks 0 and 4 with 5.0x10^8^ PFU of MAd5rhFLSC or *ΔE1B55K*rhFLSC per mouse. Two naïve mice served as controls. Spleens and blood were collected at week 6 after cervical dislocation as previously described [14].

### Intracellular cytokine staining

Splenocytes (2 × 10^6^) were treated and stained as previously described [14] except here the CD4^+^ and CD8^+^ populations, were subdivided into CD44^high^ cells. This gating groups the central memory (CM) and effector memory (EM) cells as previously described [29]. The percentage of cytokine-secreting cells in the combined memory cell subset in response to stimulation with HIV_Bal_gp120 peptides was determined following subtraction of the values obtained with non-stimulated samples.

### Antibody binding titers

Antibody binding titers were assayed by enzyme-linked immunosorbent assay (ELISA). Ninety-six well plates were coated with 1.0x10^9^ PFU Ad5hr or 100ng per well of either HIV_BaL_gp120 (ABL) or rhFLSC (Profectus BioSciences). The plates were exposed to 1% BSA blocking solution (KPL) for 2 hours at room temperature. The serum samples were serially diluted and applied in duplicate to the 96-well plates and incubated at 37°C for 1 hour. The plates were washed with PBS–Tween, exposed to either peroxidase-conjugated goat anti-mouse IgG (H + L), IgM, IgA, or rat anti-mouse IgG1 or IgG2a, and thereafter incubated for another hour. After washing the plates were developed with TMB (3, 3’, 5, 5’-tetramethylbenzidine) peroxidase substrate solution. The reaction was stopped by adding 1M H_3_PO_4_ and the plates were read at 450nm within 30 min. Endpoint titers were defined as two times the OD corresponding to the background value of the plate as the cutoff.

### Chemokine/Cytokine ELISAs

Cells were infected in serum free media, lysed with RIPA buffer (Life Technologies), and chemokines/cytokines were captured using a 25-plex Multiplex ELISA according to the manufacturer’s specifications (Life Technologies). The plates were read and analyzed using a Bio-Plex 100 instrument and software (BioRad).

### Surface staining for MIC A/B

HeLa cells were infected at a MOI of 50 for 48, 72 or 96 hours. The cells were washed twice with PBS and surface stained for 30 min at RT with an isotype control or MIC A/B-APC antibodies (Biolegend) at concentrations determined by titration, and resuspended in 1% paraformaldehyde in PBS. Approximately 20,000 cells were acquired for analysis using a FACS Calibur Cytometer. Data were analyzed using FlowJo version 9.5.2 (Tree-Star Inc.) and graphs and statistics were obtained using Prism 6.0 (GraphPad Software Inc.).

### Statistical analysis

Initial statistics were obtained using Prism v6.0 (GraphPad) and confirmed using SAS/STAT Softward version 9.3 (SAS Institute Inc.). Differences between measures were assessed using either a Mann-Whitney-Wilcoxon paired test or one-way ANOVA. Logarithmic or arcsine transformation of raw data was applied when needed for consistency with distributional assumptions.
